# Socio-demographic and regional differences in unmet healthcare needs among migrants in Europe

**DOI:** 10.1371/journal.pone.0285886

**Published:** 2023-05-18

**Authors:** Lembe Kullamaa, Rainer Reile

**Affiliations:** Department of Epidemiology and Biostatistics, National Institute for Health Development, Tallinn, Estonia; Caleb University, NIGERIA

## Abstract

**Background:**

Different barriers that hinder migrants’ access to healthcare may have detrimental effect on health but also contribute to health inequalities. Given the lack of evidence on unmet healthcare needs among European migrant population, the study aimed to analyse the demographic, socio-economic and health-related patterning of unmet healthcare needs among migrants in Europe.

**Methods:**

European Health Interview Survey data from 2013–2015 covering 26 countries was used to analyse associations of individual-level factors and unmet healthcare needs among migrants (n = 12,817). Prevalences and 95% confidence intervals for unmet healthcare needs were presented for geographical regions and countries. Associations between unmet healthcare needs and demographic, socio-economic, and health indicators were analysed using Poisson regression models.

**Results:**

The overall prevalence of unmet healthcare needs among migrants was 27.8% (95% CI 27.1–28.6) but the estimate varied substantially across geographical regions in Europe. Unmet healthcare needs due to cost or access were patterned by various demographic, socio-economic, and health-related indicators but higher prevalence of UHN were universally found for women, those with the lowest income, and poor health.

**Conclusions:**

While the high level of unmet healthcare needs illustrate migrants’ vulnerability to health risks, the regional variations in the prevalence estimates and individual-level predictors highlight the variations in national policies regarding migration and healthcare legislations and differences in welfare-systems across Europe in general.

## Introduction

Migrants, defined here as persons who change their temporary or permanent residence to or from a given country, may face barriers when trying to access healthcare services. These legal, economic, cultural, and psychological barriers can hinder migrants’ timely access to healthcare if needed and may affect their individual health but also contribute to health inequalities.

One of the main barriers is entitlement to healthcare [1–3]. In Europe, some countries have provided access to health services for migrants, especially for asylum seekers and irregular migrant children although there are disparities in access to healthcare based on legal status [4]. Even if care is formally available, its access could be hindered by several socio-economic and migration-related stressors such as low income or unemployment, social isolation, discrimination, poor living conditions and lack of health insurance [5–7]. Inability to understand the local language could prevent healthcare system access [4, 8–11] and even if migrants reach the service needed, cultural or linguistic insensitivity or negative attitudes of care staff [9] can affect the treatment quality. Previous studies have demonstrated that inefficacy in communication with healthcare workers can give rise to confidentiality issues, frustration, feeling of discrimination and disrespect, and can even lead to improper medical decisions [8–11]). Considering the evidence on cultural differences in health perceptions [11] and problems in recognising an illness or its health risks [10–12], migrant population is vulnerable in terms of health.

The concept of unmet healthcare needs (UHN) refers to difference between required and actually received health services in case of a health problem [11]. Previous estimates suggest that 26.5% of population in EU countries report UHN with most causes being related to long waiting lists and financial factors [13]. Given the evidence on barriers to healthcare access, the discrepancies between healthcare needs and access could be even more pronounced for migrant population. However, the available evidence on UHN among migrant is relatively limited. For example, an earlier study [11] has demonstrated that migration less than 5 years has shown three-fold increase in UHN as compared to non-migrants. While the differences were attenuated in time, those who migrated more than 5 years ago, still had twice the odds for UHN. Similar high levels of UHN among migrant population have been reported in a study on Korean emigrants [14] where almost half expressed UHN. Although the gap between migrants’ entitlements to care and their utilization of services is well known, there are no cross-country studies focusing on the individual-level factors that explain these differences within and between regions in Europe.

Consequently, the aim of this study was to analyse the socio-demographic patterning of UHN among migrants in Europe. In this study, we will focus on questions: a) which demographic and socio-economic indicators predict UHN due to cost and access, b) do these patterns vary when migrant’s health status is considered, and c) are these patterns different or similar across regions in Europe?

## Methods

### Data

Study uses data from the second wave of the European Health Interview Survey (EHIS 2) carried out between 2013–2015 in 31 European countries. The survey is one of the main the main statistical tools for providing harmonized and comparable data on health status, healthcare and health determinants in EU. Depending on the country, sample sizes ranged from 4001 individuals in Iceland to 25,325 individuals in Italy with either stratified or cluster sampling of individuals aged 15 and older being used in most countries. Data was collected using either face-to-face interviews or its combination with telephone interview, web questionnaire or other mode. The detailed description of survey methodology is available elsewhere [15].

### Key indicators

Migratory status is the key variable of interest. The EHIS 2 questionnaire included data on citizenship defined as i) citizen of the reporting country, ii) a citizen of another EU Member State, iii) not an EU citizen) and place of birth defined as i) native, ii) born in another EU Member State, iii) born in a non-EU country). For the current study, migrants are defined as persons who were born in and had citizenship of another (EU or non-EU) country than the country they were residing at the time of the survey (i.e., first-generation migrants). Following the study’s aim, the analytic sample was restricted to individuals that matched these criteria.

This analysis focuses on the unmet healthcare needs (UHN) due to cost or accessibility. The dependent variable was computed based on two items regarding affordability (in past 12 months could not afford i) medical examination or treatment, ii) prescribed medicines), and two items on accessibility of healthcare (unmet need for healthcare need in the past 12 months due to i) long waiting list(s), ii) distance or transportation problems). For this, individual responses to these four items were first summarized and then operationalized into binary variable with at least one positive reply defining the case of unmet healthcare need. Individuals who had not needed healthcare during the past 12 months were excluded from the analytic sample.

To analyse the regional variance in UHN, EuroVoc classification [16] was used to divide the countries into four geographical groups. Northern Europe included Denmark, Finland, Iceland, Norway, and Sweden whereas Southern Europe included Cyprus, Greece, Spain, Italy, Malta, and Portugal. Countries of Western Europe included Austria, Germany, Ireland, Luxembourg, Netherlands, and United Kingdom. Belgium (missing health variables) and France (different presentation of UHN indicators) were omitted from the analysis due to data incompatibility [17]. Central/Eastern Europe included Chechia, Croatia, Estonia, Hungary, Latvia, Lithuania, Poland, Slovak Republic, and Slovenia. As data from Bulgaria and Romania included less than 50 individuals meeting the migratory status criteria, these countries were excluded from the analysis as well.

### Demographic variables

Age was divided into six 10-year groups starting from age 20. Younger respondents were excluded from the analysis to avoid misclassification of socioeconomic status (SES) indicators. Household size was represented by the number of persons living in household categorized as a tertiary variable: i) one person; ii) 2–3 persons; iii) ≥4 persons. *De facto* cohabiting status was operationalized into binary variable with categories being i) living in a consensual union, and ii) not living in consensual union.

### Socioeconomic status

Highest level of education completed was divided in to three categories: i) tertiary, ii) secondary and/or vocational, and iii) primary education. Net monthly income of the household was presented in quintiles in ascending order with 1^st^ quintile representing the lowest and 5^th^ quintile the highest income group. Employment status was recoded into tertiary variable, i) employed, ii) unemployed, and iii) inactive, referring to students, retirees, disabled people, and other economically non-active groups.

### Health status

Health status was assessed using three items from the Minimum European Health Module [15]. Self-perceived health was categorized into tertiary variable i) good, ii) fair, or iii) bad. Long-standing health problems was a binary variable assessing whether person had suffered from any illness or health problem during the past 6 months. General activity limitation was recoded into a binary variable representing whether daily activities had been restricted due to health problems or not.

### Analytical sample and statistical analyses

The total dataset of EHIS 2 included 316,333 respondents from 30 countries. After restricting the analytical sample to migrants (n = 27,063), following exclusion criteria were used to derive analytic sample for this study: a) omitting data from countries with insufficient/incompatible data (n = 3,570), b) omitting respondents aged <20 years (n = 925), c) omitting cases with missing data on UHN (n = 1,216), d) omitting cases reporting no need for healthcare in the past 12 months (n = 8,535). The final analytical sample included thus 12,817 cases.

Descriptive statistics and Pearson’s χ^2^ test were used to describe and analyse the distribution of independent variables within analytic sample. The prevalence of UHN was calculated by dividing the number of cases reporting UHN by the total number of cases in each country/region stratum and are presented as percentages with their 95% confidence intervals. To analyse the association between independent variables and UHN, Poisson regression models with robust variance estimators were used. As odds ratios derived from logistic regression can lead to possible overestimation of the association when the outcome is frequent, the prevalence ratios (PR) derived from Poisson regression could be suitable alternative [18–20]. Following study objectives, four regression models were built. Bivariate Model 1 analyses the individual associations between independent variables and UHN. Model 2 adjusts these associations to demographic and socio-economic variables whereas model 3 adds health status variables and Model 4 also includes the variable of region. Additional analysis, stratified by region is presented for Model 3. All analyses were performed using STATA/SE 14.2 [21].

## Results

**Fig 1:**
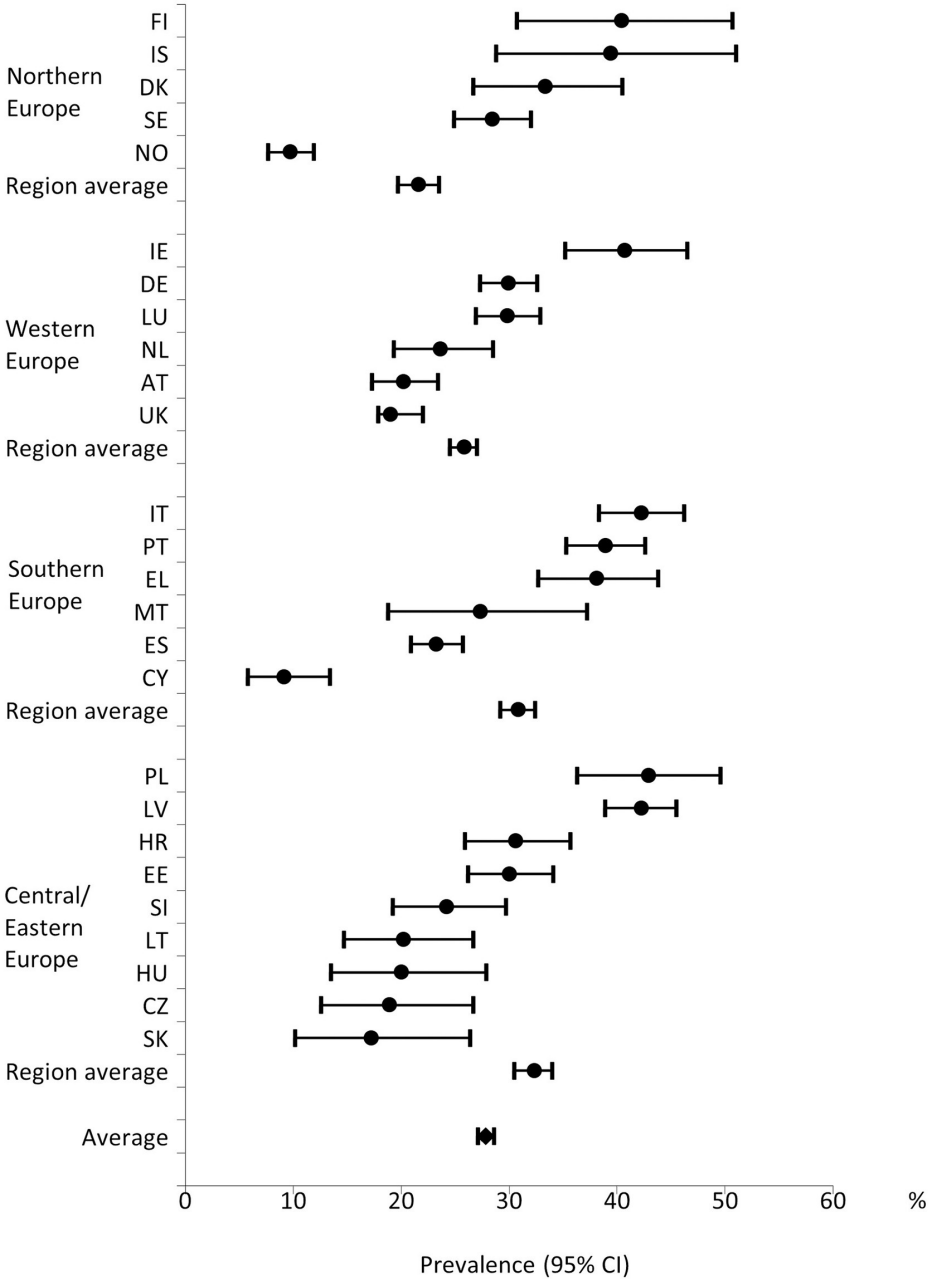
Prevalence of unmet healthcare needs with 95% CI in total foreign-born population of the study and European regions based on EHIS 2 data (2013–2015). Countries are presented on the vertical axis, and prevalence of unmet healthcare needs with 95% confidence intervals on the horizontal axis.

The overview of the analytic sample is presented in Table 1 and the prevalence of UHN by region in Fig 1. The overall prevalence of UHN among migrants was 27.8% (95% CI 27.1–28.6). By region, the prevalence varied from 21.6% (95% CI 19.7–23.5) in Northern Europe to 32.3% (95% CI 30.5–34.0%) in Central/Eastern Europe. The prevalence of UHN in Western and Southern Europe was respectively 25.8% (95% CI 24.5–27.0%) and 30.8% (95% CI 29.2–32.4%). However, considerable heterogeneity in prevalence of UHN by countries within each region was observed.

**Table 1:** General characteristics of analytic sample of migrants (n = 12,817).

Indicator	Categories	Total	Without unmet healthcare needs		Unmet healthcare needs		p-value
		N	n	%	n	%	
**Sex**	Male	5,192	3,921	42.4	1,271	35.6	< 0.000
	Female	7,625	5,329	57.6	2,296	64.4	
**Age**	20–29	1,332	972	10.5	360	10.1	0.041
	30–39	2,485	1,817	19.6	668	18.7	
	40–49	2,717	1,918	20.7	799	22.4	
	50–59	2,315	1,631	17.6	684	19.2	
	60–69	1,896	1,400	15.1	496	13.9	
	≥70	2,072	1,512	16.4	560	15.7	
**Cohabiting**	Yes	8,497	6,137	66.4	2,360	66.2	0.947
	No	4,150	2,995	32.4	1,155	32.4	
	*missing*	170	118	1.3	52	1.5	
**Household size**	1 person	2,711	1,913	20.7	798	22.4	0.103
	2–3 persons	6,553	4,752	51.4	1,801	50.5	
	≥4 persons	3,521	2,563	27.7	958	26.9	
	*missing*	*	*	*	*	*	
**Education level**	Tertiary	4,309	3,193	34.5	1,116	31.3	0.001
	Secondary/vocational	4,878	3,478	37.6	1,400	39.3	
	Primary	3,369	2,370	25.6	999	28.0	
	*missing*	261	209	2.3	52	1.5	
**Income**	5^th^ quintile	2,083	1,612	17.4	471	13.2	< 0.000
	4^th^ quintile	2,021	1,518	16.4	503	14.1	
	3^rd^ quintile	2,276	1,654	17.9	622	17.4	
	2^nd^ quintile	2,558	1,796	19.4	762	21.4	
	1^st^ quintile	2,962	2,006	21.7	956	26.8	
	*missing*	917	664	7.2	253	7.1	
**Employment status**	Employed	6,456	4,767	51.5	1,689	47.4	< 0.000
	Unemployed	1,171	773	8.4	398	11.2	
	Inactive	5,006	3,571	38.6	1,435	40.2	
	*missing*	184	139	1.5	*	*	
**Self-rated health**	Good	7,541	5,934	64.2	1,607	45.1	< 0.000
	Fair	3,660	2,442	26.4	1,218	34.2	
	Bad	1,566	837	9.1	729	20.4	
	*missing*	50	*	*	*	*	
**Long-standing health problems**	Yes	6,848	4,535	49.0	2,313	64.8	< 0.000
	No	5,913	4,674	50.5	1,239	34.7	
	*missing*	56	*	*	*	*	
**Activity limitations**	Limited	4,701	2,900	31.4	1,801	50.5	< 0.000
	Not limited	7,991	6,277	67.9	1,714	48.1	
	*missing*	125	73	0.8	52	1.5	
**Region**	Southern Europe	3,218	2,228	24.1	990	27.8	< 0.000
	Western Europe	4,930	3,660	39.6	1,270	35.6	
	Northern Europe	1,860	1,459	15.8	401	11.2	
	Central/Eastern Europe	2,809	1,903	20.6	906	25.4	

* < 50 observations, data not presented

Table 2 presents the results from regression models. In univariate analysis (Model 1), all independent variables except for respondent’s age, cohabiting status and household size were associated with UHN with prevalence ratios for UHN being higher for females, those with lower education or income levels, unemployed or non-active and those reporting poorer health or respective activity limitations. Also, regional disparities were present with prevalence ratio of UHN being statistically significantly higher for all regions compared to Northern Europe. After mutual adjustment of demographic and socio-economic variables (Model 2), the effects of sex, income and employment status were slightly attenuated and effect of education on UHN reduced to non-significant level. However, those with households of ≥4 persons had 1.16 times lower prevalence ratios of UHN compared to single-person households. After further adjustment to health variables in Model 3, all variables, except education level and cohabiting status, were statistically significantly associated with UHN. The subgroups with highest prevalence ratios of UHN were among women, those with the lowest income (1^st^ quintile), poor self-rated health, long-standing health problem and limited activity. However, compared to previous model, older age and being inactive in labour market became protective factors. Persons in the oldest age group had 1.7 times lower (PR 0.64; 95% CI 0.55–0.73, compared to 20–29-year-olds) and economically non-active persons 1.1 times lower (PR 0.91; 95% CI 0.84–0.99 compared to employed category) prevalence ratio of UHN. Inclusion of region variable had modest effect on the estimates and the pattern of association remained the same except for statistically significant effects in variables for cohabiting status where people not living in a consensual union had higher rates of UHN (PR 1.09; 95% CI 1.02–1.17).

**Table 2:** Prevalence ratios of unmet healthcare needs^a^.

Variable		Model 1^b^		Model 2^c^		Model 3^d^		Model 4^e^	
		PR	95% CI	PR	95% CI	PR	95% CI	PR	95% CI
**Sex**	Male	1		1		1		1	
	Female	**1.23**	**1.16–1.30**	**1.22**	**1.14–1.30**	**1.19**	**1.12–1.27**	**1.18**	**1.11–1.25**
**Age**	20–29	1		1		1		1	
	30–39	0.99	0.89–1.11	1.08	0.96–1.21	0.98	0.87–1.10	0.96	0.85–1.08
	40–49	1.09	0.98–1.21	**1.15**	**1.03–1.29**	0.94	0.83–1.05	0.91	0.81–1.02
	50–59	1.09	0.98–1.22	1.11	0.99–1.24	**0.80**	**0.71–0.90**	**0.78**	**0.69–0.88**
	60–69	0.97	0.86–1.09	0.96	0.84–1.09	**0.69**	**0.61–0.78**	**0.67**	**0.59–0.77**
	≥70	1.00	0.89–1.12	0.96	0.84–1.09	**0.64**	**0.55–0.73**	**0.62**	**0.54–0.71**
**Cohabiting**	Yes	1		1		1		1	
	No	1.00	0.94–1.06	1.06	0.99–1.14	1.06	0.99–1.13	**1.09**	**1.02–1.17**
**Household size**	1 person	1		1		1		1	
	2–3 persons	0.93	0.87–1.00	0.93	0.86–1.01	0.93	0.86–1.01	**0.91**	**0.84–0.99**
	≥4 persons	0.91	0.85–1.00	**0.86**	**0.78–0.94**	**0.90**	**0.82–0.99**	**0.89**	**0.81–0.97**
**Education level**	Tertiary	1		1		1		1	
	Secondary/vocational	**1.11**	**1.04–1.19**	1.07	1.00–1.15	1.00	0.93–1.08	0.98	0.81–1.05
	Primary	**1.14**	**1.06–1.23**	1.06	0.98–1.15	0.97	0.90–1.05	0.93	0.86–1.01
**Income**	5^th^ quintile	1		1		1		1	
	4^th^ quintile	1.10	0.99–1.23	1.08	0.97–1.21	1.05	0.94–1.17	1.06	0.95–1.19
	3^rd^ quintile	**1.21**	**1.09–1.34**	**1.18**	**1.06–1.31**	1.09	0.98–1.22	1.11	1.00–1.24
	2^nd^ quintile	**1.32**	**1.19–1.45**	**1.27**	**1.15–1.42**	**1.15**	**1.03**–**1.27**	**1.17**	**1.05–1.30**
	1^st^ quintile	**1.43**	**1.30–1.57**	**1.37**	**1.24–1.52**	**1.19**	**1.08**–**1.32**	**1.22**	**1.10–1.36**
**Employment status**	Employed	1		1		1		1	
	Unemployed	**1.30**	**1.19–1.42**	**1.19**	**1.08–1.31**	**1.11**	**1.01–1.23**	1.06	0.96–1.17
	Inactive	**1.10**	**1.03–1.16**	1.06	0.98–1.15	**0.91**	**0.84–0.99**	**0.91**	**0.84–0.99**
**Self-rated health**	Good	1		…		1		1	
	Fair	**1.56**	**1.47–1.66**	…		**1.35**	**1.24–1.46**	**1.34**	**1.23–1.45**
	Bad	**2.18**	**2.04–2.34**	…		**1.79**	**1.62–1.97**	**1.79**	**1.62–1.98**
**Long-standing health problems**	No	1		…		1		1	
	Yes	**1.61**	**1.52–1.71**	…		**1.22**	**1.13–1.32**	**1.18**	**1.09–1.28**
**Activity limitations**	Not limited	1		…		1		1	
	Limited	**1.79**	**1.69–1.89**	…		**1.37**	**1.27–1.49**	**1.40**	**1.29–1.51**
**Region**	Northern Europe	1		…		…		1	
	Western Europe	**1.19**	**1.08–1.32**	…		…		**1.19**	**1.07–1.33**
	Southern Europe	**1.43**	**1.29–1.58**	…		…		**1.42**	**1.28–1.59**
	Central/Eastern Europe	**1.50**	**1.35–1.66**	…		…		**1.27**	**1.13–1.43**

^a^ Statistically significant results (p < 0.05) are marked in bold

^b^ Unadjusted univariate model

^c^ Adjusted to demographic, and socioeconomic status variables

^d^ Adjusted to demographic, socioeconomic status, and health status variables

^e^ Adjusted to all variables

Model 4 also indicated that compared to Northern Europe, all other regions had significantly higher adjusted prevalence ratios of UHN. The difference was largest for Southern Europe (PR 1.42; 95% CI 1.28–1.59) followed by Central/Eastern Europe (PR 1.27; 95% CI 1.13–1.43). To analyse the potential regional variance in the associations of independent variables and UHN, Model 3 was also applied to regionally stratified data (Table 3). Results indicate that UHN were highest in all regions for women, persons with poor self-rated health, and those with illness-related activity limitations. Age was significant predictor for UHN in all regions except for Central/Eastern Europe. Especially in Northern and Western Europe, older age was a protective factor: those aged 70 years and older had 2–2.4 times lower prevalence of UHN compared to the youngest age group. Conversely, in Southern Europe, highest rates of UHN were found for ages 30–49 compared to 20–29-year-olds. The pattern differed regionally also for cohabiting status. In Northern and Central/Eastern Europe, people not living in consensual union had higher prevalence of unmet healthcare needs, yet the opposite was found for in Western Europe. Household size had significant effect on UHN only in Southern and Central/Eastern Europe where having a large household was protective factor regarding UHN. The association between socio-economic variables and UHN also varied by regions. Education was significant predictor of UHN in Western and Central/Eastern Europe, where primary education resulted in respectively 1.3 and 1.2 times lower UHN prevalence ratios compared to tertiary education. Low income was associated with UHN in Southern and Central/Eastern Europe with the former having particularly steep gradient. Employment status was significant only in Northern and Southern Europe with UHN being higher among unemployed in Northern Europe. In Southern Europe, persons inactive in the labour market had 1.2 times lower rates of UHN. While poor health and illness-related activity limitations were universally associated with higher UHN, having long-standing health problems had higher prevalence ratios of UHN in all regions except Southern Europe.

**Table 3:** Prevalence ratios of unmet healthcare needs in European regions*.

Variable		Northern Europe		Western Europe		Southern Europe		Central/Eastern Europe	
		PR	95% CI	PR	95% CI	PR	95% CI	PR	95% CI
**Sex**	Male	1		1		1		1	
	Female	**1.23**	**1.02–1.48**	**1.12**	**1.01–1.24**	**1.22**	**1.09–1.37**	**1.18**	**1.03–1.34**
**Age**	20–29	1		1		1		1	
	30–39	0.91	0.69–1.21	0.88	0.74–1.04	**1.29**	**1.03–1.61**	0.66	0.39–1.10
	40–49	0.80	0.61–1.06	**0.77**	**0.65–0.92**	**1.32**	**1.06–1.65**	0.86	0.55–1.33
	50–59	**0.68**	**0.50–0.94**	**0.68**	**0.57–0.81**	1.23	0.98–1.55	0.66	0.43–1.02
	60–69	**0.50**	**0.33–0.74**	**0.47**	**0.38–0.59**	1.13	0.86–1.47	0.75	0.49–1.15
	≥70	**0.50**	**0.32–0.77**	**0.42**	**0.33–0.54**	0.90	0.65–1.24	0.74	0.48–1.15
**Cohabiting**	Yes	1		1		1		1	
	No	**1.59**	**1.29–1.95**	**0.83**	**0.74–0.93**	1.11	0.96–1.28	**1.27**	**1.10–1.46**
**Household size**	1 person	1		1		1		1	
	2–3 persons	0.93	0.70–1.24	0.97	0.85–1.11	**0.84**	**0.73–0.96**	0.92	0.78–1.08
	≥4 persons	1.00	0.74–1.35	1.02	0.87–1.19	**0.79**	**0.67–0.92**	**0.75**	**0.59–0.96**
**Education level**	Tertiary	1		1		1		1	
	Secondary/vocational	1.17	0.94–1.46	0.93	0.83–1.04	1.11	0.96–1.29	0.91	0.79–1.04
	Primary	1.18	0.92–1.51	**0.79**	**0.68–0.92**	1.07	0.92–1.24	**0.83**	**0.70–0.98**
**Income**	5^th^ quintile	1		1		1		1	
	4^th^ quintile	0.97	0.68–1.37	0.96	0.81–1.13	1.24	0.99–1.55	1.07	0.84–1.37
	3^rd^ quintile	0.83	0.58–1.19	0.92	0.78–1.08	**1.46**	**1.17–1.81**	1.13	0.89–1.43
	2^nd^ quintile	1.02	0.73–1.42	0.88	0.74–1.04	**1.72**	**1.40–2.11**	1.11	0.88–1.41
	1^st^ quintile	1.02	0.73–1.44	0.97	0.83–1.14	**1.70**	**1.38–2.10**	**1.31**	**1.04–1.65**
**Employment status**	Employed	1		1		1		1	
	Unemployed	**1.41**	**1.05–1.90**	0.90	0.73–1.11	1.11	0.97–1.28	1.01	0.77–1.34
	Inactive	1.13	0.89–1.43	0.91	0.81–1.04	**0.84**	**0.72–0.98**	0.91	0.75–1.10
**Self-rated health**	Good	1		1		1		1	
	Fair	**1.58**	**1.20–2.06**	**1.26**	**1.11–1.44**	**1.35**	**1.18–1.54**	**1.32**	**1.07–1.64**
	Bad	**1.88**	**1.39–2.54**	**1.77**	**1.49–2.10**	**1.46**	**1.21–1.75**	**1.91**	**1.51–2.42**
**Long-standing health problem**	No	1		1		1		1	
	Yes	**1.35**	**1.02–1.78**	**1.28**	**1.13–1.45**	1.00	0.88–1.14	**1.25**	**1.00–1.56**
**Activity limitations**	Not limited	1		1		1		1	
	Limited	**1.62**	**1.23–2.15**	**1.41**	**1.24–1.60**	**1.43**	**1.25–1.63**	**1.27**	**1.06–1.52**

* Adjusted to all variables

## Discussion

Based on EHIS data from 2013–2015, the overall prevalence of UHN among migrants was 27.8%. However, the prevalence varied considerably both across and within geographical regions of Europe. UHN among migrants were also patterned by various demographic, socio-economic and health-related indicators but higher prevalence of UHN was universally found for women, those with the lowest income, poor self-rated health, and illness-related activity limitations.

Before discussing these findings in detail several aspects regarding the data and the chosen analytical approach need to be considered. EHIS is one of the few population-based cross-national studies available that includes data on UHN. As this analysis focuses on UHN among migrant sub-population, it is plausible that the underlying sample frames of national studies (and thus our analytic sample) might not represent migrant population as a whole. Refugees, undocumented migrants, temporary workers, and other similar groups are generally not included in population/dwelling registers where study samples are drawn from. Migrants might also be underrepresented due to lower response rates [22] which may also contribute to the relatively small number of migrants in EHIS 2 data. Also, as country-level data for EHIS 2 was collected over a period of time (from 2013 to 2015), the results of this study do not necessarily cover the large-scale population movements in Europe at the time of the study (e.g., Syrian refugee crisis of 2015 with only six EHIS countries’ data collection period being in 2015), nor are they directly attributable for the current situation in Europe. Also, operationalization of migrants in our study is a compromise between the complexity of the term and available data considering that birthplace and citizenship were only suitable indicators in the dataset. Without additional data, it remains unknown whether different set of indicators (e.g., current legal status, acculturation, duration of migrant status etc.) for defining migrant could have affected the results. Due to limited number of migrants in country-level data, grouping countries into regions was necessary to ensure sufficient statistical power for the analyses. Although geographical division is just one way to group European countries, we performed sensitivity analyses on other possible methods of grouping (according to GDP, and prevalence of unmet needs), but the differences in the results were marginal. Also, the country-level variations in data collection periods and methods might have introduced potential selection or response biases in migrant sub-population but these effects are attenuated in region-specific analysis. Nevertheless, the regional comparison must be approached with caution as data was not available for each country in regional clusters. Observed results do not fully represent all countries in each region, since the sample size per country varies greatly (S1 Table). Therefore, it is also not possible to make reliable one-on-one comparisons between the regions. Finally, the self-reported nature of the data needs to be considered. Although UHN items were presented in standardized questions, it includes subjective attitudes and preferences, which can be affected by the cultural and linguistic background of the respondent. Despite our effort to include a wide range of indicators, it is plausible that part of the variation in the dependent variable is described by factors that were not measured in EHIS and thus not included in the analysis.

To the best of our knowledge, this is one of the first comparative studies to focus on UHN among migrant population in Europe. Although the overall observed prevalence of UHN (27.8%) among migrants does only marginally exceed the previous estimate of 26.5% among the general population [13], these estimates varied considerably when different regions were compared. Although country- or region-specific factors were not included in current study, the relatively high prevalence of UHN and its regional variance could be at least partially explained by legislation. The right for health and access to healthcare are included in various international and European laws [3] and citizens of the EU have the right to receive emergency treatment in all EU and EEA countries when they are temporarily abroad [23]) and scheduled healthcare is available in other EU or EEA countries on the basis of home-country health insurance and reimbursement [24]. In practice, many EU countries restrict the right of non-EU migrants to healthcare, partially as an attempt to discourage further immigration [2, 3], but the differences across countries are large. Majority of European countries offer only emergency care for irregular migrants and access to both primary and specialist care is available only in the Netherlands, France, and Portugal with additional conditions on income and time of residency [4]. The UCL-Lancet Commission on Migration and Health reported that access to healthcare among authorised migrants in Europe is strongly connected to residence permits with some countries, like Bulgaria, Chechia, and Estonia, require permanent residence permits of ≥5 years to receive the same health insurance coverage as nationals [4]. These inconsistencies in healthcare availability for migrants across European countries are also one likely explanation for heterogeneity in UHN prevalence within regions. Despite this, the UHN prevalence among migrants was highest in Central/Eastern European countries, where the overall expenditures on healthcare are also among lowest in Europe [25].

The results also illustrate the demographic and socio-economic gradients in healthcare access as higher prevalence of UHN was universally found for women, unemployed, and persons with lower income. These findings are in accordance with previous studies demonstrating the link between UHN and low income [26], and the higher prevalence of UHN among women [10, 27]). It has been shown that low-income households are especially vulnerable to financial barriers in accessing healthcare due to direct (admission fees etc.), indirect (expenditures on transport etc.) or alternative costs (e.g., time away from work) [10]). While previous research has also shown that lower level of education is associated with higher prevalence of UHN [10–12]), the effects of education on UHN in our analysis were attenuated to non-significant level (in joint analysis) after adjustment to other demographic and socio-economic variables. Although education is a social determinant of health that can affect health outcomes in numerous ways [28]), it is likely that its effects are partly mediated by income variable. However, the educational differential remained significant in regional analysis for Western and Central/Eastern regions where lower education proved to be protective factor for UHN. One explanation for this somewhat unexpected finding could relate to educational bias in assessing ones’ health, as previous studies have demonstrated that people with a lower level of education may not always recognise illness or be aware of the potential health risks [10–12]). When the effects of education are considered together with lower UHN among respondents aged 50 years and older (after adjustment to health variables), the hypothesis of reporting bias receives further support as reporting bias in health at older ages and in lower education groups has previously documented [29].

Previous studies [3, 4, 11, 30] have demonstrated the importance of social support in UHN. While the aspect of formal social safety nets (health insurance and retirement pensions etc.) is also partially included in the inverse age gradient found in our data, the variables of cohabiting status and household size are more direct proxies for social support. Those who were living with partner or in households of 4 or more persons had significantly lower levels of UHN in fully adjusted model (also in Southern and Central/Eastern regions in regionally stratified analysis). While having close relationships is beneficial for health [31], its potential pathways also extend to UHN as family could provide necessary support needed to deal with the health problems. However, the effects of both indicators varied across regions. For example, having UHN were more likely for persons not living in consensual union in Northern and Central/Eastern Europe, whereas the opposite was found for Western Europe. It is plausible that differing cultural backgrounds and social norms among migrant populations in these regions could explain these variations but as the dataset did not include appropriate measures, these claims cannot be verified.

Health status itself was universally associated with UHN as persons with fair or poor self-rated health, general activity limitations and long-term health problems had higher levels of UHN. It is expected as poor overall health status indicates also higher need for healthcare services, but different barriers hinder its use. However, the question on health perception and assessment extends from subjective health outcomes to the dependent variable itself as UHN includes not just barriers to healthcare accessing but also aspects of subjective evaluation [11]. This raises the issue of how to assess UHN, which is traditionally based on subjective understanding of health problem and awareness of healthcare needs. The definition of health can depend to a large extent on a person’s education, cultural background and health-related literacy [11, 12, 26, 32]. For example, the UHN reported by people with low health literacy may not correspond to the actual number of untreated health problems [10]. Health expectations can also be shaped by differences in gender and cultural norms [33] but due to lack of relevant indicators in EHIS 2 data, no conclusions regarding cultural aspects in UHN assessment can be made.

In regional comparison, higher UHN were found for women, those with poor self-rated health and general activity limitations. Previous research [34] has described association between older age with a lower probability and higher income with higher probability of reporting UHN. Northern and Western Europe showed consistent age gradient with all groups having lower levels of UHN compared to 20–29-year-olds, while in Southern Europe the UHN were highest among 30–49 year olds. Income remained insignificant in Northern and Western regions. These may be due to differences in social care and health policies. For example, Austria and Sweden provide health insurance to authorized migrants on the same basis as to nationals [4]. It is therefore plausible, that countries in regions with longer history of migration (i.e., Northern and Western Europe) have more efficient social safety nets and strategies to address the social determinants of health of vulnerable persons [35] that mitigate the negative individual-level effects of low socio-economic status on UHN.

## Conclusion

With every fourth foreign born person in Europe reporting UHN due to cost or access to healthcare on average, the migrants are more vulnerable to health risks compared to general population. UHN among migrants were patterned by various demographic, socio-economic, and health-related indicators but higher prevalence of UHN were universally found for women, those with the lowest income, and poor health status.

Given the regional and country-level variations in UHN and its individual-level predictors, further in-depth research is needed to determine whether these differences are explained by national policies regarding migration and healthcare legislations or differences in welfare-systems across Europe in general. Moreover, as the current study period precedes current geopolitical crisis in Europe, additional study using more recent data would be necessary to validate whether the demographic and socio-economic patterns in UHN persist also after the refugee crisis related to war in Ukraine. These further analyses should also consider the heterogeneity of migrant population in terms of their legal status, socio-demographic and cultural backgrounds to provide more detailed insight into the patterns of UHN among migrants in Europe.

## Supporting information

S1 Table Final analytical sample size per country. (DOCX)
