# Supplementary material for: Socio-demographic and regional differences in unmet healthcare needs among migrants in Europe
Source: PLoS One. 2023 May 18;18(5):e0285886. doi: 10.1371/journal.pone.0285886 (PMC10194988; doi:10.1371/journal.pone.0285886)
Supplement: S1 Table — (DOCX) [file pone.0285886.s001.docx]

**S1. Final analytical sample size per country.**

| **Region** | **Country** | **Sample size (n)** |
| --- | --- | --- |
| **Northern Europe** | Denmark | 192 |
|  | Finland | 99 |
|  | Iceland | 81 |
|  | Norway | 839 |
|  | Sweden | 649 |
| **Region total:** |  | **1,860** |
| **Western Europe** | Austria | 688 |
|  | Germany | 1,167 |
|  | Ireland | 309 |
|  | Luxemburg | 936 |
|  | The Netherlands | 347 |
|  | United Kingdom | 1,483 |
| **Region total:** |  | **4,930** |
| **Southern Europe** | Cyprus | 243 |
|  | Greece | 307 |
|  | Italy | 618 |
|  | Malta | 99 |
|  | Portugal | 702 |
|  | Spain | 1,249 |
| **Region total:** |  | **3,218** |
| **Central/Eastern Europe** | Croatia | 359 |
|  | Czechia | 132 |
|  | Estonia | 546 |
|  | Hungary | 130 |
|  | Latvia | 868 |
|  | Lithuania | 188 |
|  | Poland | 224 |
|  | Slovakia | 93 |
|  | Slovenia | 269 |
| **Region total:** |  | **2,809** |
| **Total sample size:** | | **12,817** |
